# Risk factors for postoperative pulmonary complications in elderly patients undergoing video-assisted thoracoscopic surgery lobectomy under general anesthesia: a retrospective study

**DOI:** 10.1186/s12893-024-02444-w

**Published:** 2024-05-14

**Authors:** Guang Feng, Yitong Jia, Guanxu Zhao, Fanqi Meng, Tianlong Wang

**Affiliations:** Department of Anesthesiology, Xuanwu Hospital, Capital Medical University, Beijing, 100053 China

**Keywords:** Postoperative pulmonary complications, Thoracoscopic lobectomy, Risk factors, Advanced age, One-lung ventilation

## Abstract

**Background:**

The objective of this study is to identify and evaluate the risk factors associated with the development of postoperative pulmonary complications (PPCs) in elderly patients undergoing video-assisted thoracoscopic surgery lobectomy under general anesthesia.

**Methods:**

The retrospective study consecutively included elderly patients (≥ 70 years old) who underwent thoracoscopic lobectomy at Xuanwu Hospital of Capital Medical University from January 1, 2018 to August 31, 2023. The demographic characteristics, the preoperative, intraoperative and postoperative parameters were collected and analyzed using multivariate logistic regression to identify the prediction of risk factors for PPCs.

**Results:**

322 patients were included for analysis, and 115 patients (35.7%) developed PPCs. Multifactorial regression analysis showed that ASA ≥ III (*P* = 0.006, 95% CI: 1.230 ∼ 3.532), duration of one-lung ventilation (*P* = 0.033, 95% CI: 1.069 ∼ 4.867), smoking (*P* = 0.027, 95% CI: 1.072 ∼ 3.194) and COPD (*P* = 0.015, 95% CI: 1.332 ∼ 13.716) are independent risk factors for PPCs after thoracoscopic lobectomy in elderly patients.

**Conclusion:**

Risk factors for PPCs are ASA ≥ III, duration of one-lung ventilation, smoking and COPD in elderly patients over 70 years old undergoing thoracoscopic lobectomy. It is necessary to pay special attention to these patients to help optimize the allocation of resources and enhance preventive efforts.

## Introduction

Postoperative pulmonary complications (PPCs) are highly prevalent complications during the perioperative period, affecting many patients [1, 2]. These complications significantly influence patients’ outcome, prolong hospital stay, and increase perioperative mortality. PPCs, including respiratory infection, pleural effusion, respiratory failure, atelectasis, bronchospasm, pneumothorax and aspiration pneumonitis, can be caused by various factors such as patient health status, surgical trauma and anesthetic effects [3, 4].

PPCs can occur following any type of surgery, but certain surgeries carry higher risk. The incidence of PPCs is particularly high in major abdominal surgery, thoracic surgery, cardiac surgery and spinal surgery, which seriously threatens the perioperative safety of these patients [5, 6]. Previous studies have focused on these high-risk surgeries and identified several independent risk factors for PPCs. However, there still lack of study on PPCs following thoracic surgery, especially on the risk factors for PPCs after pulmonary lobectomy. It is reported that the incidence of PPCs in thoracic surgery can be as high as 20 ∼ 50%, posing a significant threat to patients undergoing high-risk thoracic surgeries [7, 8]. Identifying and mitigating these risk factors is essential for improving outcomes in thoracic patients.

Pulmonary lobectomy is a common surgical treatment for lung diseases such as lung cancer and emphysema. In thoracic surgery, lobectomy involves a large resection range and significant trauma, which can have substantial impacts on pulmonary function [9]. In recent years, video-assisted thoracoscopic surgery (VATS) has been widely applied due to its advantages such as less tissue trauma and enhanced postoperative recovery [10, 11]. However, PPCs are still common following VATS lobectomy. These complications range from mild atelectasis and pneumonia to severe respiratory failure and sepsis, posing a considerable threat to the perioperative safety of patients [5]. Previous studies have shown that advanced age is one of the independent risk factors for PPCs [12]. This may be related to the age-related decline in pulmonary function and immune responsiveness in elderly patients [13]. Age-related physiological changes can lead to a decrease in pulmonary function, weakened immune response, reduced tolerance to surgery, and increased risk of PPCs [14]. Therefore, it is particularly important to assess the risk factors for PPCs in elderly patients who undergo thoracoscopic lobectomy and take appropriate measures to reduce the occurrence of these complications.

There is limited study on the risk factors for PPCs in elderly patients undergoing pulmonary lobectomy. The aim of this study is to investigate the risk factors associated with PPCs in elderly patients aged over 70 years old who have undergone VATS lobectomy. By identifying these risk factors, we hope to provide clinicians with a better understanding of the important risks involved in these patients and to guide the development of tailored prevention and management strategies to improve patients’ postoperative recovery.

## Methods

### Study design

This retrospective observational study of cohorts reviewed the medical records of 322 patients aged ≥ 70 years old who underwent VATS lobectomy under general anesthesia between January 1, 2018 and August 31, 2023. The patients were selected from a larger database of thoracic surgery patients at Xuanwu Hospital, Capital Medical University. This study was approved by the ethics committee of Xuanwu Hospital, Capital Medical University (2022 No.028). Since this is a retrospective study, informed consent from patients is not required.

### Study population

To ensure the accuracy and reliability of the study, the inclusion and exclusion criteria were established. The inclusion criteria included patients aged ≥ 70 years old who underwent elective thoracoscopic lobectomy under general anesthesia. The exclusion criteria excluded patients with pre-existing pulmonary complications such as lung infections, atelectasis, or pleural effusion before surgery, as well as those who had undergone chest surgery within 30 days before surgery. Patients who underwent conversion from VATS to open thoracotomy during the operation were excluded. Additionally, patients with missing important clinical data and those who underwent emergency surgery were also excluded.

### Outcome definition

The primary outcome variable for our analysis was PPCs within 7 days after surgery. The diagnostic criteria for PPCs are not exactly the same in different studies. In our study, the definition of PPCs is based on the European Perioperative Clinical Outcome (EPCO) definitions, which were jointly developed by the European Anesthesiology Society and the European Society of Intensive Care Medicine in 2015 [4]. The EPCO standard defines PPCs as the composite outcome of different pulmonary complications that occur after surgery, including respiratory infection, respiratory failure, pleural effusion, atelectasis, pneumothorax, bronchospasm and aspiration pneumonitis. These complications can lead to a significant increase of perioperative mortality in surgical patients. The EPCO definitions provide a standardized approach to the identification and classification of PPCs, allowing for more consistent and comparable data collection in clinical study and practice.

The preoperative assessment of cardiac function for patients is conducted using the New York Heart Association (NYHA) Functional Classification. The length of hospital stay (LOS) was recorded on days, from the date of hospitalization to the date of discharge. The net intraoperative fluid infusion volume refers to the difference between the total amount of infused fluids and the total amount of excreted fluids during surgery. In this study, the net intraoperative fluid infusion volume is calculated by subtracting the sum of blood loss and urine output from the total intraoperative fluid infusion. The mortality rate in this study was calculated as the death rate during hospitalization. The postoperative analgesia in this study refers to the use of intravenous analgesia pumps for patients, utilizing Patient-Controlled Intravenous Analgesia method. The drug formula and dosage for postoperative analgesia were determined by different anesthetists. The evaluation of postoperative analgesia effect was conducted by anesthesia nurses in the ward on the first day after surgery, using the Visual Analog Scale (VAS) score for patients.

### Data collection

Data on patient basic characteristics, preoperative laboratory tests, surgical information, anesthesia information, intraoperative conditions, preoperative systemic comorbidities and postoperative outcomes were collected from the electronic medical records. Based on the previous relevant studies and our clinical experience, we collected potential risk factors to be evaluated, including gender, age, American Society of Anesthesiologists (ASA) classification, surgical approach, body mass index (BMI), preoperative laboratory indices, smoking history, drinking history, duration of operation, duration of one-lung ventilation (OLV), blood loss, and transfusion requirements, postoperative analgesia, history of respiratory disease and other systemic diseases [12, 15, 16].

### Statistical analysis

The statistical software used for data analysis in this study is SPSS, version 23.0. Continuous variables were expressed as mean ± standard deviation or median (interquartile range, IQR) depending on whether they adhere to a normal distribution. Categorical variables were expressed as the number and proportion. Patients were divided into two groups depending on whether they developed PPCs. Differences in continuous parameters were calculated using Student’s t-test or Mann-Whitney U-test as appropriate. Comparisons of categorical variables were performed using chi-square test or Fisher’s exact test as appropriate. *P* < 0.05 was considered statistically significant. The statistically significant variables from the univariate analysis were further analyzed using the multivariate logistic regression to determine the independent predictors of PPCs.

## Results

**Fig. 1:**
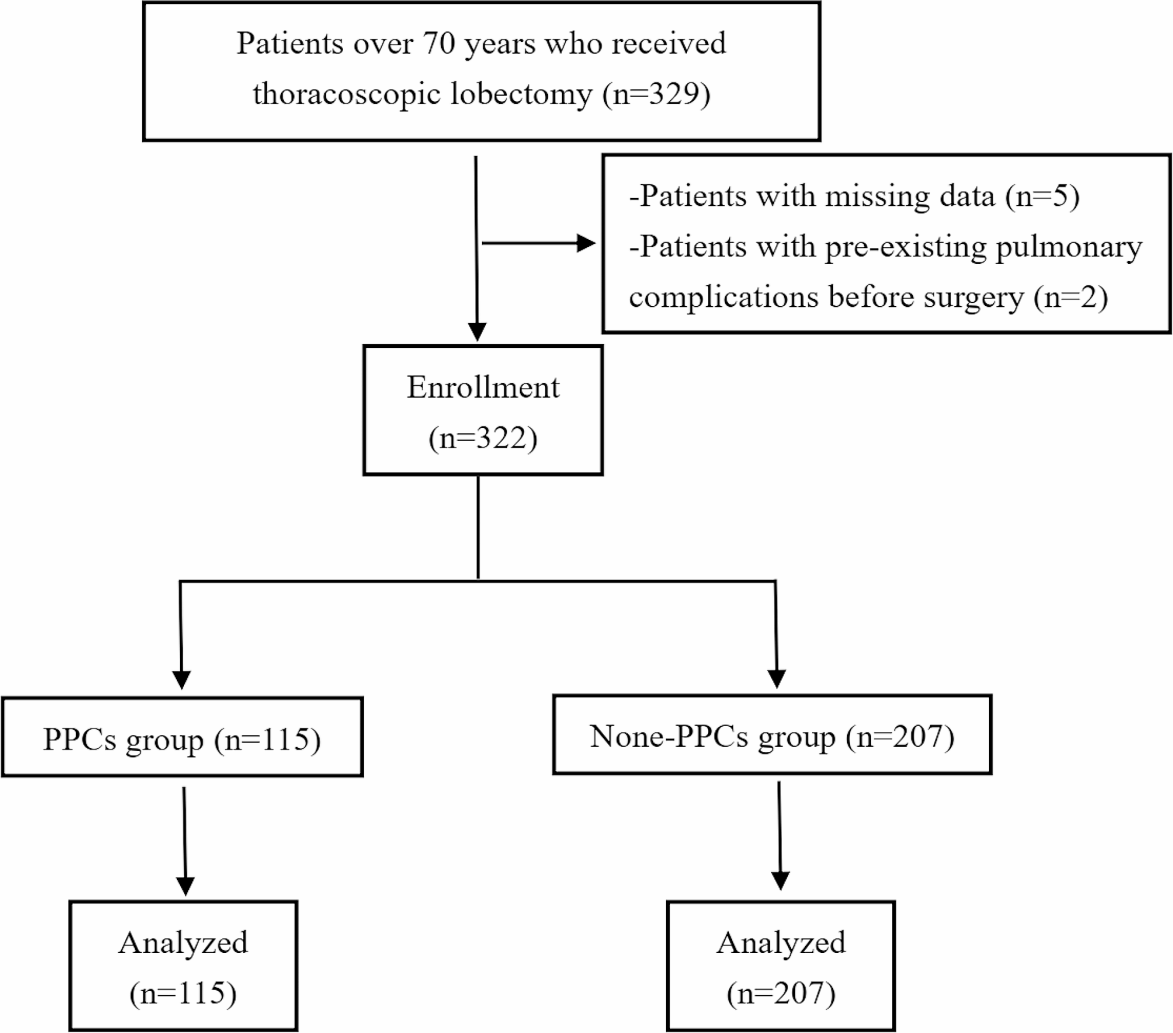
Flow diagram of the study populations

The study included 322 patients, comprising 189 males and 133 females, with ages ranging from 70 to 84 years old. Detailed patient characteristics are shown in Table 1. The results showed that there were 189 documented PPCs in 115 patients which developed PPCs (Fig. 1; Table 2). As shown in Table 2, among these patients, respiratory infections were the most common PPC, accounting for 68.7% (79/115), followed by pleural effusion at 52.2% (60/115). Other PPCs included atelectasis at 22.6% (26/115), pneumothorax at 13.0% (15/115), respiratory failure at 7.0% (8/115) and bronchospasm at 0.9% (1/115). No case of aspiration pneumonia was found.

**Table 1:** Univariate analysis of patients’ characteristics and PPCs

Characteristics	PPCs group *n* = 115, (%)	None-PPCs group *n* = 207, (%)	*P*-value
Age, years (median, IQR)	73(72,76)	73(71,75)	0.194
Sex			0.288
male	72(62.6)	117(56.5)	
female	43(37.4)	90(43.5)	
BMI			0.266
< 28	96 (83.5)	182 (87.9)	
≥ 28	19 (16.5)	25 (12.1)	
ASA classification			0.001
<III	33(28.7)	100(48.3)	
≥III	82(71.3)	107(51.7)	
NYHA classification			0.598
<III	106(92.2)	194(93.7)	
≥III	9(7.8)	13(6.3)	
Preoperative hemoglobin (g/L)			0.203
Hb ≥ 110	109(94.8)	188(90.8)	
Hb < 110	6(5.2)	19(9.2)	
Preoperative albumin (g/L)			0.730
ALB ≥ 35	99(86.1)	181(87.4)	
ALB < 35	16(13.9)	26(12.6)	
Duration of operation (hour)	3.37(2.70, 4.47)	2.72(2.15, 3.47)	<0.001
Duration of one-lung ventilation (hour)	3.00(2.25, 4.00)	2.33(1.83, 3.00)	<0.001
Net intraoperative fluid infusion volume (ml)	990(500,1300)	880(500,1250)	0.286
Intraoperative blood loss (ml)			0.004
< 200	92(80.0)	189(91.3)	
≥ 200	23(20.0)	18(8.7)	
Intraoperative blood transfusion			0.145
No	107(93.0)	200(96.6)	
Yes	8(7.0)	7(3.4)	
Lesions			
Benign	5(4.3)	12(5.8)	0.577
Malignant	110(95.7)	195(94.2)	
Number of operated lung lobes			0.015
Single lung lobe	100 (87)	196(94.7)	
Multiple lung lobes	15 (13)	11(4.3)	
Smoking			0.001
No	63 (54.8)	151 (72.9)	
Yes	52 (45.2)	56 (27.1)	
Drinking			0.064
No	85 (73.9)	171 (82.6)	
Yes	30 (26.1)	36 (17.4)	
Anesthesia method			0.203
Intravenous anesthesia	109 (94.8)	188 (90.8)	
Intravenous combined with respiration	6 (5.2)	19 (9.2)	
Hypertension			0.325
No	56(48.7)	89(43.0)	
Yes	59(51.3)	118(57.0)	
Diabetes mellitus			0.092
No	95(82.6)	154(74.4)	
Yes	20(17.4)	53(25.6)	
Coronary artery disease			0.103
No	85(73.9)	169(81.6)	
Yes	30(26.1)	38(18.4)	
Myocardial infarction			0.365
No	108(93.9)	199(96.1)	
Yes	7(6.1)	8(3.9)	
Chronic bronchitis			0.007
No	85(73.9)	178(86.0)	
Yes	30(26.1)	29(14.0)	
COPD			0.002
No	103(89.6)	202(97.6)	
Yes	12(10.4)	5(2.4)	
Asthma			0.338
No	110(95.7)	202(97.6)	
Yes	5(4.3)	5(2.4)	
Postoperative analgesia			0.222
No	21(18.3)	50(24.2)	
Yes	94(81.3)	157(75.8)	
VAS score			0.126
VAS < 4	82(87.2)	146(93.0)	
VAS ≥ 4	12(12.8)	11(7.0)	

Note: PPCs, postoperative pulmonary complications; BMI, body mass index; ASA, American Society of Anesthesiologists; NYHA, New York Heart Association; COPD, chronic obstructive pulmonary disease; VAS, visual analog scale

**Table 2:** Frequency of postoperative pulmonary complications

PPCs	Number of patients (%)
Respiratory infection	79(68.7)
Pleural effusion	60(52.2)
Atelectasis	26(22.6)
Pneumothorax	15(13.0)
Respiratory failure	8(7.0)
Bronchospasm	1(0.9)
Total Number of Patients with one or more PPCs	115(35.7)

Note: PPCs, postoperative pulmonary complications

Patients were divided into two groups depending on the occurrence of PPCs. As shown in Table 3, we conducted the clinical outcome analysis and found that the median LOS in the PPCs group was 15 (11, 19) days, significantly longer than that of the None-PPCs group, which was 12 (9, 15) days (*P* < 0.001). Furthermore, there were 3 death cases in the PPCs group, while there was no death case in the None-PPCs group. There was significant difference in perioperative mortality between the PPCs group and the None-PPCs group during hospitalization (*P* = 0.02, Table 3).

**Table 3:** Patients’ outcomes

Outcomes	PPCs group *n* = 115, (%)	None-PPCs group *n* = 207, (%)	*P*-value
Death	3(2.6)	0(0.0)	0.020
LOS (day)	15(11,19)	12(9,15)	<0.001

Note: PPCs, postoperative pulmonary complications; LOS, length of hospital stay

The variables that are related to the occurrence of PPCs in the univariate analysis are presented in Table 1. The following eight variables, such as ASA classification, duration of operation, duration of OLV, intraoperative blood loss, number of operated lung lobe, smoking, chronic bronchitis and COPD showed statistical differences between the PPCs group and the None-PPCs group. There were no significant statistical differences between the PPCs group and the None-PPCs group in terms of gender, age, NYHA classification, diabetes mellitus, hypertension, preoperative hemoglobin, preoperative albumin, coronary artery disease, myocardial infarction, infusion volume, blood transfusion, drinking and anesthesia method.

The multivariate logistic regression analysis was performed to ascertain the independent risk factors for PPCs following thoracoscopic lobectomy. Based on the results of univariate analysis, eight variables such as ASA classification, duration of operation, duration of OLV, intraoperative blood loss, smoking, number of operated lung lobes, chronic bronchitis and COPD were included in the multivariate analysis. As shown in Table 4, multivariate analysis identified four independent risk factors for PPCs after thoracoscopic lobectomy, which are ASA ≥ III, duration of OLV, smoking and COPD.

**Table 4:** Multivariate logistic analysis of independent risk factors for postoperative pulmonary complications

Parameters	OR	95% CI	*P*-value
ASA classification	2.084	1.230 ∼ 3.532	0.006
Duration of operation (hour)	0.784	0.382 ∼ 1.612	0.508
Duration of OLV (hour)	2.281	1.069 ∼ 4.867	0.033
Intraoperative blood loss (ml)	0.901	0.368 ∼ 2.206	0.820
Smoking	1.850	1.072 ∼ 3.194	0.027
Number of operated lung lobe	2.329	0.922 ∼ 5.884	0.074
Chronic bronchitis	1.771	0.910 ∼ 3.443	0.092
COPD	4.274	1.332 ∼ 13.716	0.015

Note: ASA, American Society of Anesthesiologists; OLV, one-lung ventilation; COPD, chronic obstructive pulmonary disease

## Discussion

In the retrospective study of patients over 70 years old who underwent VATS lobectomy, we found that the incidence of PPCs among the 322 patients was 35.7%. Patients who developed PPCs had significantly longer LOS and higher perioperative mortality rate. The study further identified four independent risk factors for PPCs following thoracoscopic lobectomy, which are ASA ≥ III, duration of OLV, smoking and COPD.

The common surgical procedures for lungs in clinical practice include, but are not limited to, lobectomy, segmentectomy and wedge resection. These surgical procedures are primarily utilized to treat various diseases in lungs or thoracic cavity, such as lung cancer, tuberculosis and bronchiectasis. Lobectomy, in particular, is a relatively common thoracic surgical procedure. However, lobectomy carries a higher level of trauma compared to segmentectomy and wedge resection [17]. For thoracic surgery, PPCs are the most common complications that pose a threat to patients’ perioperative safety and affect their prognosis. Previous study has identified advanced age as a significant risk factor for the development of PPCs [18, 19]. Specifically, elderly patients who undergo more invasive surgical procedures like lobectomy are at a heightened risk of experiencing PPCs. Based on this, the current study aimed to identify the risk factors for PPCs in patients over 70 years old who have undergone thoracoscopic lobectomy, in order to identify measures to improve the postoperative recovery quality of these high-risk groups.

In this study, there were no deaths in the None-PPCs group, while there were three deaths in the PPCs group. The detailed information are as follows: Death case 1: A male patient died on the first day after the surgery due to respiratory failure and heart failure. Death case 2: A male patient developed pneumothorax and respiratory failure after the surgery and died on the eighth day postoperatively due to respiratory failure. Death case 3: A female patient died suddenly on the second day after the surgery due to acute hypoxemia and respiratory failure. The direct cause of death in all three patients was respiratory failure, which may have been caused by different underlying factors. This underscores the critical importance of perioperative monitoring of pulmonary function in patients undergoing lung lobectomy. For patients with high risks, more detailed examinations and assessments should be conducted, and necessary preoperative optimization treatments should be administered. Postoperatively, it is essential to closely monitor patients’ vital signs and promptly identify and manage early complications that may affect cardiopulmonary function.

The ASA classification is a widely recognized indicator for predicting surgical risks. The ASA classification assigns patients ranging from I to IV based on their health status and underlying disease conditions by evaluating their physical condition before surgery. The higher ASA classification indicates the higher risk of surgery. Several previous studies have shown the significant correlation between ASA classification and the risk of PPCs [15, 20]. Most opinions hold that when the ASA grade reaches III or above, the risk of patients developing PPCs significantly increases. The current study also found the same conclusion that the risk of PPCs significantly increases in patients with ASA classification greater than III. However, other studies have failed to confirm the association between ASA classification and PPCs [21]. This may be due to differences in the study population and surgical type in different studies.

In recent years, obesity has become one of the major global epidemics, affecting the health of individuals worldwide. Previous studies have shown that obesity is closely associated with PPCs [22, 23]. Obese patients often face greater surgical challenges and have poorer postoperative recovery of cardiopulmonary function [24]. According to the WHO standards for Asians, BMI ≥ 28 is considered obese. However, there was no significant statistical difference in the incidence of PPCs between the BMI ≥ 28 group and the BMI < 28 group in the current study. The possible reasons may be that the sample size is still relatively small, or there are differences in the surgical types and the studied population. Clinically, obese patients may have the increased risk of PPCs due to impaired oxygen exchange function, elevated risk of hypoxemia and delayed recovery of cardiopulmonary function. Therefore, preoperative assessment and preparation for obese patients are very importance, with a specific focus on maintaining pulmonary function during the perioperative period.

In previous studies, smoking has been identified as a significant risk factor for the development of PPCs [25, 26]. This study also confirms this point. The harmful substances present in tobacco, including tar and nicotine, have the potential to cause damage to the airway epithelium, leading to a reduction in the airway’s ability to clear debris effectively. This damage results in an increase in mucus production and a decrease in lung compliance, ultimately compromising pulmonary function. Furthermore, smokers exhibit shorter and irregularly shaped cilia on the bronchial epithelium, which impairs mucus clearance and predisposes them to postoperative pulmonary infections [27]. Previous study has shown that patients who continue to smoke within two weeks prior to surgery are at a significantly increased risk of developing postoperative pulmonary infections compared to those who quit smoking at least two weeks prior to surgery [26]. It suggests that smoking cessation prior to surgery may be a feasible strategy to reduce the risk of PPCs and improve outcomes following thoracic surgery. Unfortunately, the detail information relating whether smoking patients had quitted smoking before surgery could not be obtained in our retrospective electronic records. Prospective studies remain to be conducted to further determine the correlation of smoking cessation and PPCs for smoking patients.

Previous studies have investigated the duration of operation and found that longer operation time is closely related to the increase of pulmonary complications [3, 28]. In addition to studying the duration of operation, this study also includes the duration of OLV intraoperatively. The results showed that the duration of operation and the duration of OLV were significantly associated with PPCs in univariate analysis. However, multivariate analysis showed that only the duration of OLV was the independent risk factor for PPCs, while the duration of operation was not. Since the main surgical steps of pulmonary lobectomy are performed under OLV, it seems to indicate that the longer duration of OLV is one of the main causes of intraoperative potential lung injury and PPCs. OLV is a commonly used lung isolation technique in thoracic surgery. OLV often leads to the significant increase in airway pressure and excessive ventilation pressure. Prolonged higher-pressure ventilation time may cause lung contusion, edema, exacerbation of inflammatory reactions and even cause pulmonary hemorrhage. Besides, previous studies have shown that continuous OLV during surgery produces a large amount of oxygen free radicals and the longer the operation time, the longer the OLV time, and the more oxygen free radicals produced [29]. In addition, the potential damage of OLV includes hypoxemia, atelectasis and pulmonary edema [30]. The quality and duration of intraoperative OLV are mainly related to the anesthetic and surgical techniques. Therefore, it is important to develop a rigorous and meticulous surgical plan before surgery and strengthen team cooperation to minimize the duration of OLV. Pulmonary protection strategies during OLV are also topics that require further exploration in the future.

Previous studies have shown that the effect of postoperative analgesia is correlated with the occurrence of PPCs [31]. Moderate to severe postoperative pain may lead to respiratory restrictions for patients. The pain may make patients unwilling to take deep breaths or cough, which may result in the retention of pulmonary secretions, increasing the risk of atelectasis and pulmonary infection. However, there was no statistically significant difference in postoperative analgesia and VAS scores between the PPCs group and the None-PPCs group in the univariate analysis of this study. One of the possible reasons is that the surgeons have administered additional analgesic drugs to relieve their pain for most patients with moderate to severe pain. Patients who experienced moderate to severe pain on the first day after surgery mostly received good pain management subsequently.

In this study, we collected preoperative pulmonary comorbidities including chronic bronchitis, COPD and asthma. The results of regression analysis showed that COPD is one of the independent risk factors for PPCs. This is consistent with previous studies [32, 33]. Elderly patients over 70 years old who undergo thoracic surgery often have multiple comorbidities. The results demonstrate that pulmonary comorbidities have the closest relationship with PPCs compared to other system comorbidities. This is primarily because patients with pulmonary comorbidities may have poorer baseline pulmonary function. Additionally, surgical trauma, anesthesia and mechanical ventilation can all exacerbate ventilatory and gas exchange dysfunction, as well as affect airway mucosal secretions and expectoration [13]. COPD is a prevalent respiratory disorder that is typically marked by persistent airflow limitation, with main symptoms including chronic cough, sputum production and dyspnea. Due to the impaired pulmonary function in patients with COPD, they face the higher risk of PPCs following surgical procedures [34]. Therefore, there is a close relationship between COPD and PPCs. For patients with COPD, preoperative evaluation and postoperative management are crucial.

There are certain limitations of this study. Firstly, this study is a retrospective study conducted in a single center with a relatively small sample size (322 cases), some uncertainty may exist. Secondly, the time and severity of PPCs were not further stratified. The observation indicators of this study were PPCs within 7 days, while short-term pulmonary complications after discharge were not included. This may affect the statistical analysis of the incidence and risk factors of PPCs. The time and severity of pulmonary complications are important for patient prognosis. We can further collect the detailed time and severity of PPCs for deeper analysis in the future. Thirdly, in terms of case data collection, due to the hospital’s electronic case system constraints, some important clinical examination indicators were not fully collected, so they were not discussed in this study. Further conclusions require multi-center, prospective clinical studies for verification.

## Conclusions

In summary, PPCs in elderly patients (≥ 70 years old) who undergo VATS lobectomy are affected by multiple factors. The present study has identified ASA ≥ III, duration of OLV, smoking and COPD as potential independent risk factors for PPCs. The analysis of risk factors for PPCs in high-risk individuals provides valuable and reliable indicators for assessing the risk of these complications. This information can assist in identifying high-risk patients in clinical practice and taking targeted preparations and interventions before surgery to proactively prevent PPCs. It is particularly important to perform rigorous and cautious preoperative assessments, personalized perioperative management and multidisciplinary comprehensive treatment for these high-risk patients.

## Data Availability

The datasets used and/or analyzed during the current study are available from the corresponding author on reasonable request.

## Abbreviations

PPCs: Postoperative pulmonary complications
VATS: Video-assisted thoracoscopic surgery
EPCO: European Perioperative Clinical Outcome
COPD: Chronic obstructive pulmonary disease
NYHA: New York Heart Association
LOS: Length of hospital stay
VAS: Visual analog scale
ASA: American Society of Anesthesiologists
BMI: Body mass index
OLV: One-lung ventilation
